# Postoperative autotransfusion drain after total hip arthroplasty: a meta-analysis of randomized controlled trials

**DOI:** 10.1038/srep27461

**Published:** 2016-07-01

**Authors:** Hui Xie, Jian-Ke Pan, Kun-Hao Hong, Da Guo, Jian Fang, Wei-Yi Yang, Jun Liu

**Affiliations:** 1 Second School of Clinical Medicine, Guangzhou University of Chinese Medicine, Guangzhou 510405, China; 2 Department of Orthopaedics, Second Affiliated Hospital of Guangzhou University of Chinese Medicine (Guangdong Provincial Hospital of Chinese Medicine), Guangzhou 510120, China; 3 Department of Orthopaedics, Guangdong Second Traditional Chinese Medicine Hospital, Guangzhou 510095, China; 4 Department of Orthopaedics, Third Affiliated Hospital of Guangzhou University of Traditional Chinese Medicine, Guangzhou 510375, China

## Abstract

The use of a postoperative autotransfusion drain (PATD) to reduce allogenic blood transfusions in total hip arthroplasty (THA) remains controversial. Therefore, we conducted a meta-analysis to evaluate the efficacy and safety of this technique. Randomized controlled trials (RCTs) were identified from PubMed, Embase, and the Cochrane Central Register of Controlled Trials (CENTRAL). Thirteen RCTs (1,424 participants) were included in our meta-analysis. The results showed that PATD reduced the rate of allogenic transfusions (RR = 0.56; 95% CI [0.40, 0.77]) and total blood loss (MD = −196.04; 95% CI [−311.01, −81.07]). Haemoglobin (Hb) levels were higher in the PATD group on postoperative day 1 (MD = 0.28; 95% CI [0.06, 0.49]), but no significant differences on postoperative days 2 or 3 (MD = 0.29; 95% CI [−0.02, 0.60]; MD = 0.26; 95% CI [−0.04, 0.56]; respectively). There were no differences in length of hospital stay (MD = −0.18; 95% CI [−0.61, 0.25]), febrile reaction (RR = 1.26; 95% CI [0.95, 1.67]), infection (RR = 0.95; 95% CI [0.54, 1.65]), wound problems (RR = 1.07; 95% CI [0.87, 1.33]), or serious adverse events (RR = 0.59; 95% CI [0.10, 3.58]). Our findings suggest that PATD is effective in reducing the rate of allogenic transfusion. However, the included studies are inadequately powered to conclusively determine the safety of this technique.

Total hip arthroplasty (THA) is accompanied by substantial blood loss, averaging 1,000–2,000 ml^1^,^2^,^3^ and a decline of 3.0 to 4.0 g/dl in haemoglobin levels^3^. Moreover, hidden blood loss can account for 60% of total blood loss, ranging from 612 to 1,603 ml^4^. This substantial blood loss potentially contributes to delayed postoperative rehabilitation, a longer hospital stay, and even mortality. Thus, patients undergoing THA typically require transfusion. However, with an increased awareness of the potential deleterious effects of allogenic blood transfusion, including infection, transfusion-associated lung injury and circulatory overload, and mortality^5^,^6^,^7^,^8^,^9^,^10^, a consensus has emerged on perioperative blood management that allogenic blood transfusion should be minimized. Nevertheless, the rate of allogenic blood transfusions remains high due to the growing number of THA procedures^1^,^11^. Saleh *et al.* stated that the increase in allogenic transfusion is associated with increased complications, longer hospital stays, and increased cost. Thus, they recommended the effective utilization of blood conservation methods^1^.

Autologous blood transfusion, including preoperative autologous blood donation, intraoperative blood salvage and postoperative autotransfusion drain (PATD), is considered effective in reducing allogenic blood transfusion and its underlying risks^9^,^12^,^13^. In several autologous transfusions, PATD is considered relatively simple to implement and potentially cost-effective^14^,^15^. Such drainage devices collect postoperatively shed blood and then retransfuse the shed blood (washed or unwashed) to patients within 6 hours postoperatively. Previous studies have demonstrated that PATD significantly reduces the rate of allogenic transfusion and results in reduced blood loss^16^,^17^,^18^,^19^,^20^. However, the use of the PATD remains controversial, and some studies have questioned its effectivenss^21^,^22^,^23^,^24^,^25^. To resolve the existing uncertainties, we performed a meta-analysis to evaluate the efficacy and safety of PATD compared with a closed-suction drain (CSD).

## Results

### Study selection

**Figure 1:**
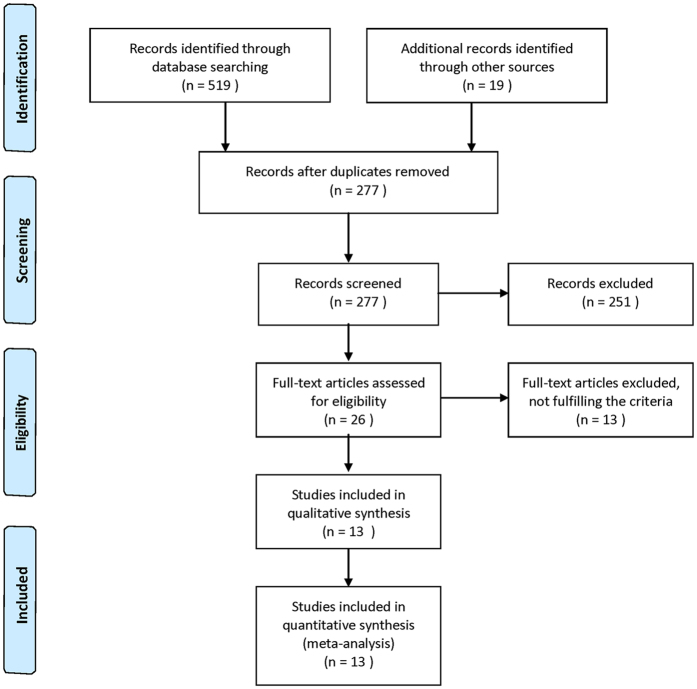
Flow diagram of the study selection.

A total of 277 records were searched via database and manual searches. After a thorough screening of titles and abstracts, 251 records were excluded. The remaining 26 articles were assessed in a full-text review. Finally, thirteen studies^16^,^17^,^18^,^21^,^22^,^26^,^27^,^28^,^29^,^30^,^31^,^32^,^33^ involving 1,424 participants met the inclusion criteria and were included in the meta-analysis (Fig. 1).

### Characteristics of included studies

The characteristics of the included studies are listed in Table 1. Eight studies performed only primary THA^16^,^17^,^18^,^21^,^22^,^27^,^28^,^29^, one study performed only revision surgery^30^, and the remaining studies performed both. Four studies involved total knee arthroplasty^18^,^28^,^31^,^33^; however, data related to THA were extracted. In one three-arm study^31^, two different postoperative autotransfusion devices were compared with CSD. We combined these two autotransfusion groups according to the Cochrane Handbook^34^.

### Risk of bias

**Figure 2:**
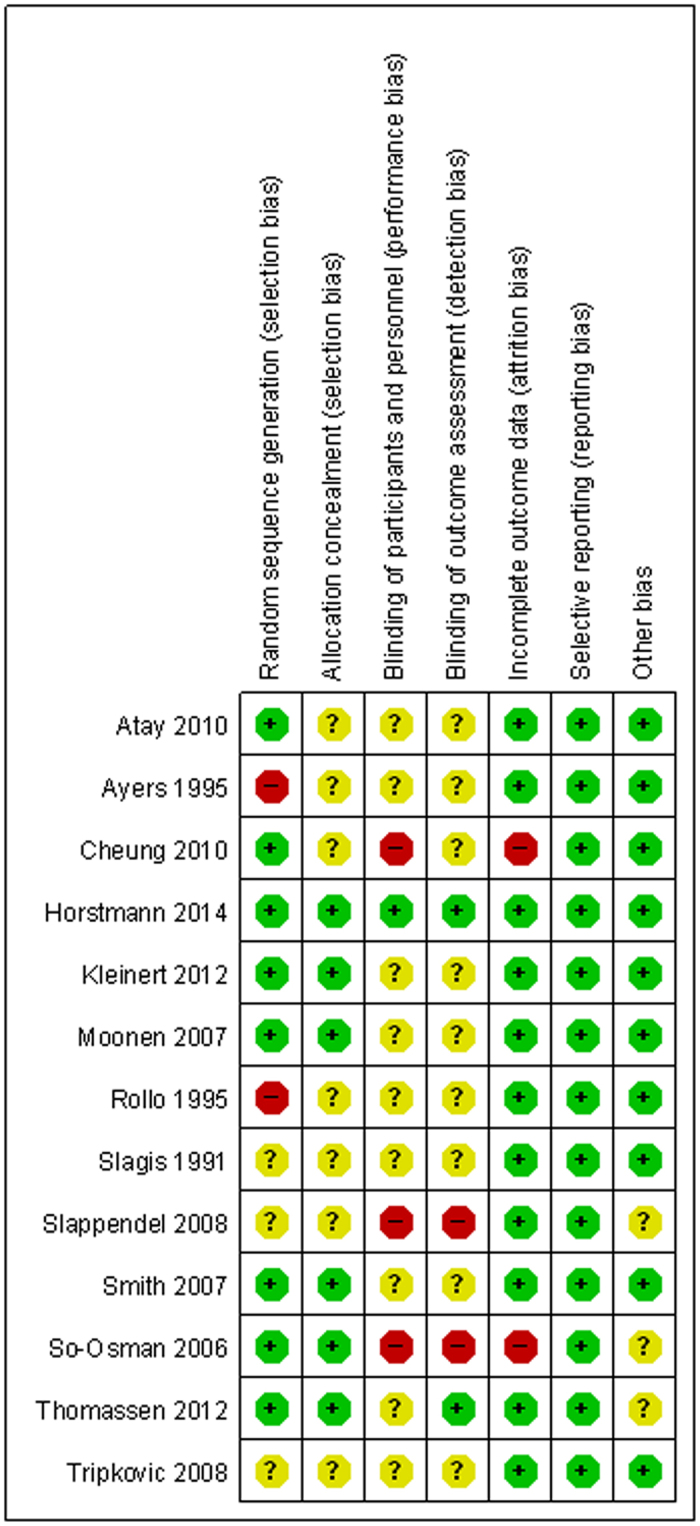
Summary of risk of bias of included RCTs. “+” represents low risk of bias; “?” represents unclear risk of bias; “−” represents high risk of bias.

The assessment of risk of bias is shown in Fig. 2. Random sequence generation was mentioned in all included studies. Ten of the studies detailed the methods of randomization used^16^,^17^,^18^,^21^,^22^,^26^,^27^,^28^,^31^,^32^; however, two used inadequate randomization (one was randomized by month of birth^22^, and another was randomized by hospital number^32^), which led to categorization as “high risk”. Six studies described adequate allocation concealment^16^,^17^,^18^,^21^,^26^,^31^. Only two studies described the blinding methods used: one performed double blinding of surgeons and assessors^16^, and another performed blinding of the study assessors^26^. Two studies had a high risk of incomplete outcome data^27^,^31^ due to a lack of details in some adverse events. In addition, we categorized three studies as of unclear risk based on other biases due to funding from device manufacturers^26^,^30^,^31^.

### Outcomes of the meta-analysis

All data regarding transfusion rate, total blood loss, postoperative Hb, length of hospital stay, febrile reaction, infection, wound problems and serious adverse events were pooled for comparison. The overall outcomes are summarized in Table 2.

### Rate of allogenic transfusion

**Figure 3:**
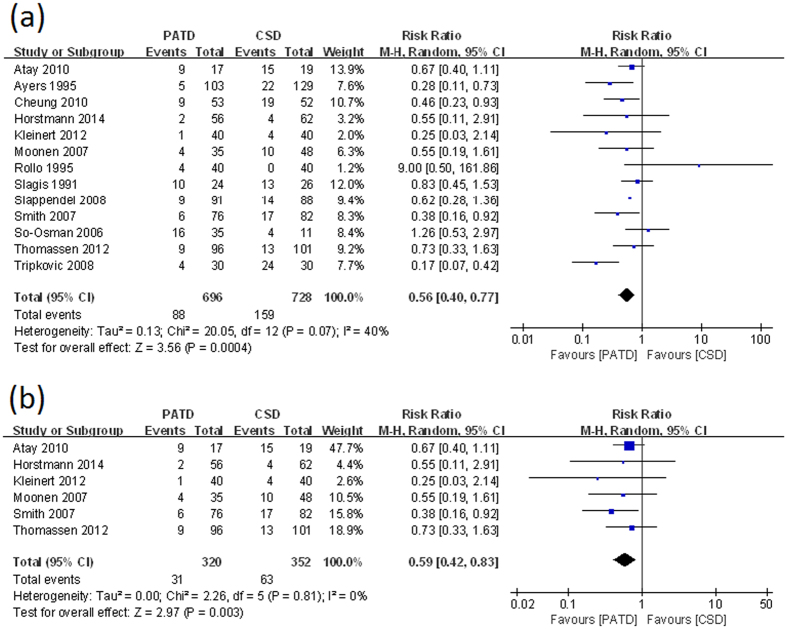
(**a**) Forest plot and meta-analysis of allogenic transfusion rate in all included studies. (**b**) Forest plot and meta-analysis of allogenic transfusion rate in high-quality studies.

All thirteen studies^16^,^17^,^18^,^21^,^22^,^26^,^27^,^28^,^29^,^30^,^31^,^32^,^33^ reported the rate of allogenic transfusion; the data from these studies were pooled. The pooled results showed that PATD significantly reduced the rate of allogenic transfusion (RR = 0.56; 95% CI: 0.40 to 0.77; p = 0.0004; Fig. 3a), with a small to moderate heterogeneity (p = 0.07, I^2^ = 40%). Moreover, when only high-quality studies were pooled, the result showed the same effect in the PATD group (RR = 0.59; 95% CI: 0.42 to 0.83; p = 0.003; Fig. 3b), with no significant heterogeneity (p = 0.81, I^2^ = 0%).

### Total blood loss

**Figure 4:**
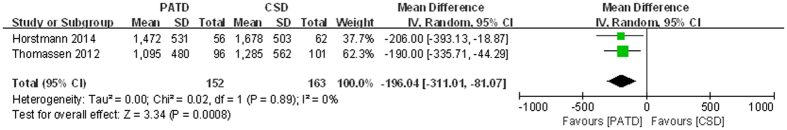
Forest plot and meta-analysis of total blood loss.

Data regarding total blood loss were only available in two studies^16^,^26^. No significant heterogeneity was found (p = 0.89, I^2^ = 0%). The pooled results showed that total blood loss was lower in patients treated with PATD (MD = −196.04; 95% CI: −311.01 to −81.07; p = 0.0008; Fig. 4).

### Postoperative haemoglobin level

**Figure 5:**
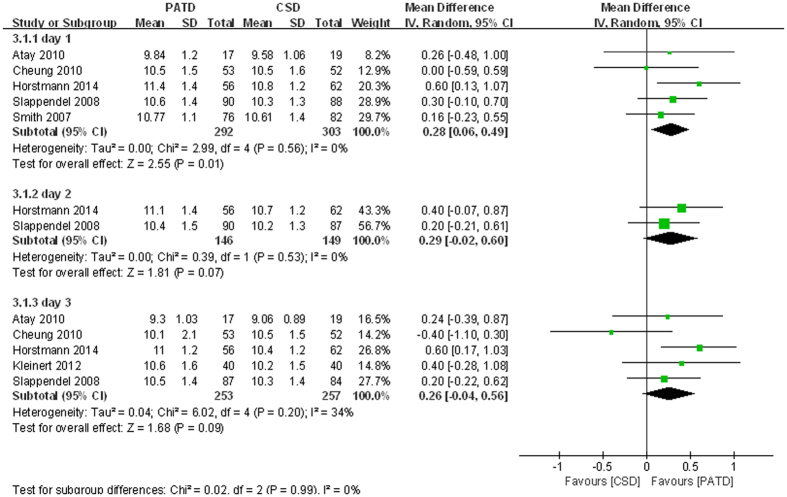
Forest plot and meta-analysis of postoperative Hb levels.

Six studies^16^,^17^,^21^,^27^,^28^,^30^ reported the Hb levels on days 1–3 after surgery. Therefore, we performed subgroup meta-analyses to compare the Hb levels based on the date. There were no significant heterogeneities among the subgroups (p = 0.56, I^2^ = 0%; p = 0.53, I^2^ = 0%; p = 0.2, I^2^ = 34%; respectively). On the first postoperative day, the PATD group maintained a higher level (MD = 0.28; 95% CI: 0.06 to 0.49; p = 0.01; Fig. 5). However, there were no significant differences between the two groups on postoperative days 2 or 3 (MD = 0.29; 95% CI: −0.02 to 0.60; p = 0.07; MD = 0.26; 95% CI: −0.04 to 0.56; p = 0.09; respectively; Fig. 5).

### Length of hospital stay

**Figure 6:**
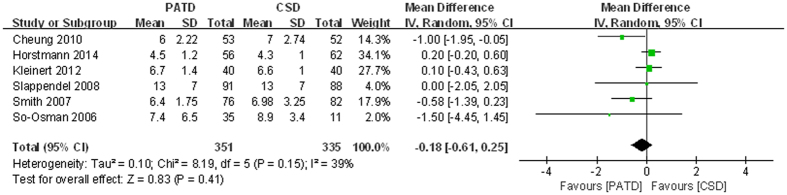
Forest plot and meta-analysis of length of hospital stay.

Six studies^16^,^17^,^21^,^27^,^30^,^31^ reported the length of hospital stay. There was no difference between the two groups (MD = −0.18; 95% CI: −0.61 to 0.25; p = 0.41; Fig. 6), and heterogeneity was low (p = 0.15, I^2^ = 39%).

### Febrile reaction

**Figure 7:**
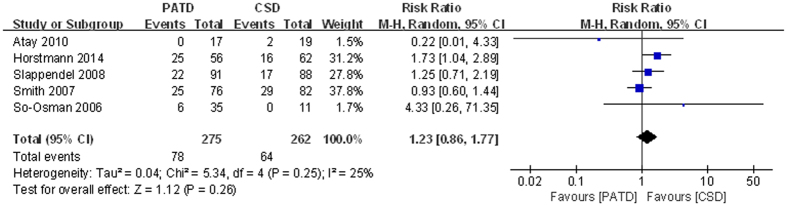
Forest plot and meta-analysis of febrile reaction.

Five studies^16^,^17^,^28^,^30^,^31^ reported febrile reactions. No significant difference was observed between the two groups (RR = 1.26; 95% CI: 0.95 to 1.67; p = 0.11; Fig. 7), and heterogeneity was low (p = 0.25, I^2^ = 25%).

### Infection

**Figure 8:**
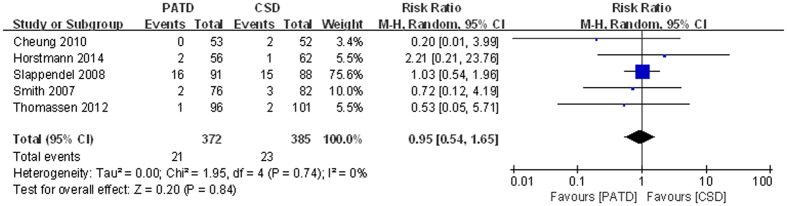
Forest plot and meta-analysis of infection.

Infections were documented in five studies^16^,^17^,^26^,^27^,^30^. The pooled results showed no significant differences between the two groups in terms of infection (RR = 0.95; 95% CI: 0.54 to 1.65; p = 0.84; Fig. 8); no significant heterogeneity was observed (p = 0.74, I^2^ = 0%).

### Wound problems

**Figure 9:**
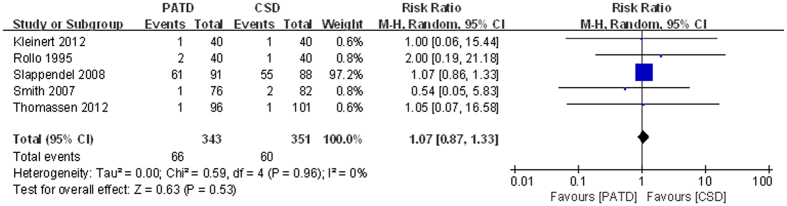
Forest plot and meta-analysis of wound problems.

Wound problems were reported in five studies^17^,^21^,^22^,^26^,^30^. The two groups did not differ significantly (RR = 1.07; 95% CI: 0.87 to 1.33; p = 0.53; Fig. 9), and no significant heterogeneity was observed (p = 0.96, I^2^ = 0%).

### Serious adverse events

**Figure 10:**
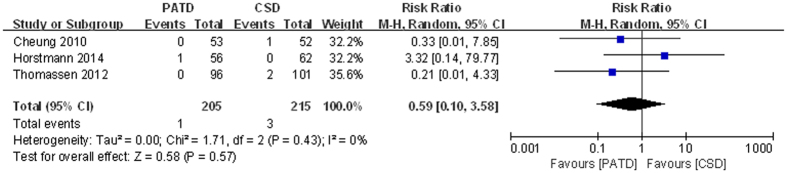
Forest plot and meta-analysis of serious adverse events.

Only three studies^16^,^26^,^27^ reported serious adverse events, including one death in the PATD group and one pulmonary embolism and two deaths in the CSD group. No significant heterogeneity was observed (p = 0.43, I^2^ = 0%). No significant difference was found between the two groups (RR = 0.59; 95% CI: 0.10 to 3.58; p = 0.57; Fig. 10).

### Sensitivity analysis

Sensitivity analysis was performed by removing each study individually to identify whether the pooled results changed. All results were stable except postoperative Hb levels. On postoperative day 1, the difference between groups became statistically insignificant after the removal of one study^16^; in addition, the removal of another study^27^ on postoperative day 3 reduced I^2^ to 0% but resulted in a significant difference between groups. In addition, two studies^22^,^29^ accounted for the main source of heterogeneity of the allogenic transfusion rate; removing these two studies resulted in a large reduction of heterogeneity (I^2^ decreased to 0%); however, the results still suggested that PATD reduced the transfusion rate.

### Publication bias

Publication bias was evaluated using Begg’s test and Harbord’s test (or Egger’s test). There was no evidence for significant publication bias among most of the included studies. Details are shown in Table 3.

## Discussion

Although restrictive blood management and several transfusion alternatives have been developed for minimizing exposure to allogenic blood^35^,^36^,^37^,^38^,^39^,^40^,^41^, an increasing rate of allogenic transfusion remains following THA due to a number of identifiable risk factors, such as female gender, older age, black race, medical insurance and previous anaemia^1^,^5^,^42^,^43^. Moreover, under certain circumstances, allogenic transfusion is not feasible, such as with Jehovah’s Witnesses who refuse allogenic blood and patients with rare blood types. Optimizing the use of blood conservation potentially resolves such conditions; nevertheless, there is little evidence regarding the efficacy of PATD.

Previous meta-analyses^12^,^20^,^24^,^44^ have investigated the efficacy and safety of cell salvage in THA. However, the strength of these meta-analyses was weakened by poor methodological quality or other limitations, and the conclusions were inconsistent. The studies by Carless *et al.* and Haien *et al.* combined several types of orthopaedic surgery, which inevitably resulted in clinical heterogeneity because a tourniquet is commonly used in total knee arthroplasty. Moreover, most included studies had a high risk of bias. In Li *et al.*’s meta-analysis^24^, PATD showed no effect on reducing the transfusion rate but appeared to be associated with less total blood loss and lower superficial infection. However, few studies were included to analyse the transfusion rate as well as certain other outcomes. In a more recent study^44^, inconsistent results were obtained; when all studies were pooled, the conclusion favoured cell salvage, but the pooled results of recent trials (2010 to 2012) showed no difference between groups. Because the authors subjectively considered that studies published after 2010 had a lower risk of bias, the subgroup analyses appeared to explain the clinical significance and the substantial heterogeneity in other subgroups with difficulty. Furthermore, the analysis might have neglected some high-quality trials published before 2010 or included recent trials of poor quality. In addition, the authors only compared the transfusion rate; other data of clinical significance were not analysed. Given the defects found in previous studies, we performed the present meta-analysis to determine whether PATD could be of greater benefit to THA patients than CSD. We eliminated potential confounding factors from total knee arthroplasty and intraoperative cell salvage, which controlled for the clinical heterogeneity of the included studies. Moreover, the results of high-quality studies strengthened the conclusion.

To our knowledge, the current study is the largest meta-analysis that has independently investigated the use of PATD after THA. Thirteen eligible RCTs, including 1,424 participants, were included in our meta-analysis to evaluate the efficacy and safety of PATD.

Overall, the most important findings were that PATD significantly reduces the rate of allogenic blood transfusion. PATD is associated with a 44% reduction in the exposure rate of allogenic blood. Moreover, the pooled results of high-quality studies showed a similar effect (a 41% reduction of RR) with no significant heterogeneity (I^2^ = 0, p = 0.81). A recent large cohort study investigated 2,087,423 patients undergoing THA who received allogenic transfusion. The results showed that allogenic transfusion was associated with a longer hospital stay, increased costs, and worse surgical and medical outcomes^1^. Another study analysed data from more than 12,000 patients who underwent THA or total knee arthroplasty. That study also demonstrated that allogenic transfusion was significantly associated with a higher risk of infection^10^. Therefore, the reduction of allogenic transfusion could potentially decrease the risk of numerous comorbidities and total costs. Although different transfusion management strategies may affect the transfusion rate, in the high-quality studies, restrictive transfusion triggers were used and the transfusion rate was still reduced, indicating that PATD provided an independent effect; this conclusion is strengthened by the methodological quality.

Increased allogenic transfusion is associated with increased blood loss^45^. Total blood loss was shown to be lower in the PATD group, a finding that might account for the lower allogenic transfusion rate. Nonetheless, only two studies reported the calculated blood loss, which considers hidden blood loss^3^,^4^; thus, it is difficult to draw a conclusion due to the small number of included studies. Other studies reporting estimated blood loss were not available to pool the data.

Although transfusion decisions should consider various factors, haemoglobin concentration remains an important indicator^46^,^47^. A higher postoperative Hb level is correlated with a lower transfusion rate, better early functional recovery, and higher patient satisfaction^16^,^48^. In this meta-analysis, we found that the PATD group maintained a higher Hb level on the first postoperative day, but no differences were present on the next two days. A potential explanation for this finding is that PATD was only used within 6 hours after surgery. However, this result changed when subjected to sensitivity analysis, suggesting that it was unstable. Munoz *et al.* indicated that no increase in a patient’s Hb levels should be expected due to the lower haemoglobin concentration in postoperatively salvaged blood. Indeed, the retransfusion of shed blood is likely to maintain Hb levels above the transfusion trigger until bleeding stops^15^.

Regarding length of hospital stay, several studies reported a shorter length of hospital stay in the PATD group^14^,^49^,^50^. However, no difference was observed in our study. Given that the length of hospitalization might be correlated with a number of confounding factors, such as patient rehabilitation, comorbidities, and different discharge policies, we recommend that future studies describe the standard of discharge with more details and isolate the potential confounders.

Postoperative shed blood, particularly unwashed blood, may be contaminated with wound material and contains a variety of tissue materials and chemical debris, potentially causing complications, such as febrile reaction, infections, embolism, immune response and even death^25^,^51^,^52^. Washed shed blood is considered safer because most bioactive contaminants are removed^53^,^54^,^55^; however, washing shed blood is expensive and complex. Nevertheless, studies have suggested that the incidence of adverse events is lower than theoretically predicted^15^,^51^,^56^. In addition, with recent improvements in techniques and practices, the use of cell salvage is safe, even in obstetrics or malignancy^13^. Similarly, in our study, adverse events showed a low incidence. However, no differences in adverse events between PATD and CSD were observed. This finding may be due to the low incidence; most of the included studies were underpowered to accurately reflect the incidence of adverse events. Therefore, future studies with larger sample sizes are urgently needed to determine the safety of PATD.

In spite of the rigorous protocol of this meta-analysis, several limitations should be taken into account. First, some of the included studies had one or more risks of bias, such as inappropriate randomization, no allocation concealment, lack of blinding, and other shortcomings, which limited the reliability of the outcomes. However, the pooled results of high-quality studies reached the same conclusion, which strengthens our conclusion. Second, the number of studies investigating several outcomes was relatively small because some data were not available; thus, these outcomes may be changed by the findings of future research. Third, there were several transfusion triggers in different studies, which might be a potential source of clinical heterogeneity that affects the results. In addition, the sample sizes of most of the included studies did not have sufficient power to draw a conclusion regarding adverse events, suggesting that further study on this topic is needed.

In conclusion, we found that PATD is effective in reducing the rate of allogenic transfusion in patients undergoing THA; a reduction of RR of more than 40% was found. Moreover, the use of PATD appears to be associated with a higher postoperative Hb level and less total blood loss, without any significant adverse events. Thus, PATD may reduce the exposure to allogenic blood and its underlying risks. The current evidence may guide clinicians in their decisions with regard to transfusion for THA patients. However, due to the limitations of this meta-analysis, we recommend more widely accepted transfusion guidelines, and additional well-designed RCTs with adequate sample sizes and a consolidated standard are needed.

## Methods

### Search strategy

A comprehensive search was performed in the PubMed, Embase, and Cochrane Central Register of Controlled Trials databases up to March 2016. The search terms included “autologous blood transfusion”, “operative blood salvage”, “autotransfusion”, “blood salvage”, “retransfusion”, “arthroplasty, replacement, hip”, “total hip arthroplast*”, “total hip replacement*”, and “total hip prosthes*”. The related references in the identified studies were manually searched.

### Selection criteria

The inclusion criteria were as follows: (1) randomized controlled trial; (2) patients treated with primary or revision total hip arthroplasty; (3) PATD compared with CSD; (4) at least one of the key data available, including allogenic transfusion rate, total blood loss, Hb level, length of hospital stay, infection, febrile reaction, wound problems or serious adverse events.

The exclusion criteria were as follows: (1) duplicate articles; (2) cohort studies, case reports, editorials, letters, reviews, and animal experimental studies; (3) data that could not be extracted.

### Data extraction

Two reviewers (HX and JKP) independently extracted the following data from the included studies: authors’ names, date of publication, sample size, patients’ age and gender, surgery type, allogenic transfusion rate, total blood loss, preoperative and postoperative Hb levels, length of hospital stay, febrile reaction, infection rate, wound problems (wound leakage, haematoma, delayed healing) and severe adverse events (life-threatening events and death). In the event of missing data, we attempted to contact the corresponding authors for details.

### Quality assessment

The methodological quality of the included studies was independently evaluated by two reviewers (HX and HKH) using the Cochrane Collaboration’s tool for assessing the risk of bias^34^. These domains were selection bias (random sequence generation and allocation concealment), performance bias (blinding of participants and personnel), detection bias (blinding of outcome assessments), attrition bias (incomplete outcome data), reporting bias (selective reporting) and other bias (other sources of bias). Any disagreements were resolved by discussion or were arbitrated by the corresponding author (JL).

### Statistical analysis

Risk ratio (RR) and mean difference (MD) were used to pool dichotomous and continuous data, respectively. The meta-analysis was performed using Review Manager 5.3.5 (Cochrane Collaboration, Oxford, UK). For continuous data presented as the mean with quartile/range, the standard deviations were estimated according to the Cochrane Handbook^34^ or the method described by Hozo *et al.*^57^. Heterogeneity was assessed using the Cochrane Q test and I-square statistic. A sensitivity analysis was performed to identify the source of the heterogeneity. All data were pooled using the random-effects model. Begg’s test and Harbord’s test (or Egger’s test) were used to estimate potential publication bias.

## Additional Information

**How to cite this article**: Xie, H. *et al.* Postoperative autotransfusion drain after total hip arthroplasty: a meta-analysis of randomized controlled trials. *Sci. Rep.* **6**, 27461; doi: 10.1038/srep27461 (2016).

## Figures and Tables

**Table 1:** Characteristics of the included studies.

Author	Date	Sample size		Gender (F/M)		Age (Y)		Preoperative Hb level (g/dL)		Types of surgery
		PATD	CSD	PATD	CSD	PATD	CSD	PATD	CSD	
Atay^28^	2010	17	19	6/11	6/13	59.76 ± 15.43	58.95 ± 13.6	13.52 ± 1.07	12.98 ± 1.46	P
Ayers^32^	1995	103	129	125/107		72 (20 to 89)		12.9	12.9	P & R
Cheung^27^	2010	53	52	39/22	30/24	65 (61 to 73)	70.5 (63 to 76)	13.6 (13.0 to 14.4)	13.7 (12.7 to 14.3)	P
Horstmann^16^	2014	56	62	36/20	42/20	67.6 ± 9.1	69.3 ± 9.5	14.2 ± 1.3	14.1 ± 0.9	P
Kleinert^21^	2012	40	40	19/21	19/21	66 ± 10	64 ± 11	14.2 (11.4 to 17.1)	14.0 (10.2 to 16.6)	P
Moonen^18^	2007	35	48	NA	NA	NA	NA	NA	NA	P
Rollo^22^	1995	40	40	16/24	20/20	68 (28 to 87)	64 (39 to 85)	NA	NA	P
Slagis^33^	1991	24	26	NA	NA	NA	NA	NA	NA	P & R
Slappendel^30^	2008	91	88	54/37	56/32	68 ± 10	69 ± 11	13.8 ± 1.4	13.9 ± 1.4	R
Smith^17^	2007	76	82	40/36	42/40	73.5 (52 to 87)	75.5 (46 to 91)	13.61 (9.3 to 17.1)	13.59 (10.3 to 16.5)	P
So-Osman^31^	2006	35	11	NA	NA	NA	NA	NA	NA	P & R
Thomassen^26^	2012	96	101	69/27	65/36	67 ± 11	65 ± 12	13.87 ± 1.16	13.98 ± 1.16	P & R
Tripkovic^29^	2008	30	30	16/14	18/12	68 ± 12	71 ± 11	NA	NA	P

PATD: postoperative autotransfusion drain; CSD: closed-suction drain; F: female; M: male; Y: years; P: primary arthroplasty; R: revision arthroplasty; NA: data not available.

**Table 2:** Summary of meta-analysis outcomes.

Outcomes	N	Patients(PATD/CSD)	Overall effect		Heterogeneity	
			RR or MD (95% CI)	P	I^2^	P
Transfusion rate						
All included studies	13	696/728	0.56 [0.40, 0.77]	0.0004	40%	0.07
High-quality studies	6	320/352	0.59 [0.42, 0.83]	0.003	0%	0.81
Total blood loss	2	152/163	−196.04 [−311.01, −81.07]	0.0008	0%	0.89
Postoperative Hb						
Day 1	5	292/303	0.28 [0.06, 0.49]	0.01	0%	0.56
Day 2	2	146/149	0.29 [−0.02, 0.60]	0.07	0%	0.53
Day 3	5	253/257	0.26 [−0.04, 0.56]	0.09	34%	0.2
Hospital stay	6	351/335	−0.18 [−0.61, 0.25]	0.41	39%	0.15
Febrile reaction	5	275/262	1.26 [0.95, 1.67]	0.11	25%	0.25
Infections	5	372/385	0.95 [0.54, 1.65]	0.84	0%	0.74
Wound problems	5	343/351	1.07 [0.87, 1.33]	0.53	0%	0.96
Serious adverse events	3	205/215	0.59 [0.10, 3.58]	0.57	0%	0.43

N: number of studies; RR: risk ratio; MD: mean difference; CI: confidence interval.

**Table 3:** Assessment of publication bias.

Outcomes	N	Begg’s test	Harbord’s test or Egger’s test
Allogenic transfusion rate			
All included studies	13	p = 0.583	p = 0.35
High-quality studies	6	p = 0.26	p = 0.247
Total blood loss	2	p = 1.000	NA*
Postoperative Hb			
Day 1	5	p = 1.000	p = 0.852
Day 2	2	p = 1.000	NA*
Day 3	5	p = 0.806	p = 0.378
Length of hospital stay	6	p = 0.260	p = 0.137
Febrile reaction	5	p = 1.000	p = 0.926
Infection	5	p = 0.221	p = 0.388
Wound problems	5	p = 0.806	p = 0.907
Serious adverse events	3	p = 0.296	p = 0.616

N: number of studies; NA: data not available; *Assessment of publication bias could not be performed because the number of studies was less than 3.

## References

[b1] Saleh A. *et al.* Allogenic Blood Transfusion Following Total Hip Arthroplasty: Results from the Nationwide Inpatient Sample, 2000 to 2009. J Bone Joint Surg Am 96A, e155 (2014). 25232085 10.2106/JBJS.M.00825 PMC4159964

[b2] Borgen P. O., Dahl O. E. & Reikeras O. Blood loss in cemented THA is not reduced with postoperative versus preoperative start of thromboprophylaxis. Clin Orthop Relat Res 470, 2591 (2012). 22476844 10.1007/s11999-012-2320-9 PMC3830101

[b3] Sehat K. R., Evans R. L. & Newman J. H. Hidden blood loss following hip and knee arthroplasty. Correct management of blood loss should take hidden loss into account. J Bone Joint Surg Br 86, 561 (2004). 15174554

[b4] Liu X. *et al.* Hidden blood loss after total hip arthroplasty. J Arthroplasty 26, 1100 (2011). 21256705 10.1016/j.arth.2010.11.013

[b5] Hart A. *et al.* Blood transfusion in primary total hip and knee arthroplasty. Incidence, risk factors, and thirty-day complication rates. J Bone Joint Surg Am 96, 1945 (2014). 25471908 10.2106/JBJS.N.00077

[b6] Li G. *et al.* Incidence and transfusion risk factors for transfusion-associated circulatory overload among medical intensive care unit patients. Transfusion 51, 338 (2011). 20723173 10.1111/j.1537-2995.2010.02816.x PMC3006039

[b7] Li G. *et al.* Long-term survival and quality of life after transfusion-associated pulmonary edema in critically ill medical patients. Chest 137, 783 (2010). 19837827 10.1378/chest.09-0841 PMC2851555

[b8] Marik P. E. & Corwin H. L. Efficacy of red blood cell transfusion in the critically ill: a systematic review of the literature. Crit Care Med 36, 2667 (2008). 18679112 10.1097/CCM.0b013e3181844677

[b9] Newman E. T. *et al.* Impact of perioperative allogeneic and autologous blood transfusion on acute wound infection following total knee and total hip arthroplasty. J Bone Joint Surg Am 96, 279 (2014). 24553883 10.2106/JBJS.L.01041

[b10] Friedman R., Homering M., Holberg G. & Berkowitz S. D. Allogeneic blood transfusions and postoperative infections after total hip or knee arthroplasty. J Bone Joint Surg Am 96, 272 (2014). 24553882 10.2106/JBJS.L.01268

[b11] Kurtz S., Ong K., Lau E., Mowat F. & Halpern M. Projections of primary and revision hip and knee arthroplasty in the United States from 2005 to 2030. J Bone Joint Surg Am 89, 780 (2007). 17403800 10.2106/JBJS.F.00222

[b12] Carless P. A. *et al.* Cell salvage for minimising perioperative allogeneic blood transfusion. Cochrane Database Syst Rev D1888 (2010). 10.1002/14651858.CD001888.pub4 PMC4163967 20393932

[b13] Ashworth A. & Klein A. A. Cell salvage as part of a blood conservation strategy in anaesthesia. Br J Anaesth 105, 401 (2010). 20802228 10.1093/bja/aeq244

[b14] Markar S. R., Jones G. G., Karthikesalingam A., Segaren N. & Patel R. V. Transfusion drains versus suction drains in total knee replacement: meta-analysis. Knee Surg Sports Traumatol Arthrosc 20, 1766 (2012). 22072326 10.1007/s00167-011-1761-0

[b15] Munoz M., Slappendel R. & Thomas D. Laboratory characteristics and clinical utility of post-operative cell salvage: washed or unwashed blood transfusion? Blood Transfus 9, 248 (2011). 21084005 10.2450/2010.0063-10 PMC3136591

[b16] Horstmann W. G. *et al.* Favourable results of a new intraoperative and postoperative filtered autologous blood re-transfusion system in total hip arthroplasty: A randomised controlled trial. Int Orthop 38, 13 (2014). 24077886 10.1007/s00264-013-2084-1 PMC3890134

[b17] Smith L. K., Williams D. H. & Langkamer V. G. Post-operative blood salvage with autologous retransfusion in primary total hip replacement. J Bone Joint Surg Br 89, 1092 (2007). 17785752 10.1302/0301-620X.89B8.18736

[b18] Moonen A. F., Knoors N. T., van Os J. J., Verburg A. D. & Pilot P. Retransfusion of filtered shed blood in primary total hip and knee arthroplasty: a prospective randomized clinical trial. Transfusion 47, 379 (2007). 17319816 10.1111/j.1537-2995.2007.01127.x

[b19] Strumper D. *et al.* Clinical efficacy of postoperative autologous transfusion of filtered shed blood in hip and knee arthroplasty. Transfusion 44, 1567 (2004). 15504161 10.1111/j.1537-2995.2004.03233.x

[b20] Haien Z., Yong J., Baoan M., Mingjun G. & Qingyu F. Post-operative auto-transfusion in total hip or knee arthroplasty: a meta-analysis of randomized controlled trials. Plos One 8, e55073 (2013). 23372816 10.1371/journal.pone.0055073 PMC3555861

[b21] Kleinert K., Werner C., Mamisch-Saupe N., Kalberer F. & Dora C. Closed suction drainage with or without re-transfusion of filtered shed blood does not offer advantages in primary non-cemented total hip replacement using a direct anterior approach. Arch Orthop Trauma Surg 132, 131 (2012). 21874574 10.1007/s00402-011-1387-1

[b22] Rollo V. J., Hozack W. J., Rothman R. H., Chao W. & Eng K. O. Prospective randomized evaluation of blood salvage techniques for primary total hip arthroplasty. J Arthroplasty 10, 532 (1995). 8523015 10.1016/s0883-5403(05)80157-3

[b23] Benjamin J. B. & Colgan K. M. Are Routine Blood Salvage/Preservation Measures Justified in All Patients Undergoing Primary TKA and THA? J Arthroplasty (2015). 10.1016/j.arth.2015.01.031 25662674

[b24] Li N., Li P., Liu M., Wang D. & Xia L. Comparison between autologous blood transfusion drainage and no drainage/closed-suction drainage in primary total hip arthroplasty: a meta-analysis. Arch Orthop Trauma Surg 134, 1623 (2014). 25288027 10.1007/s00402-014-2090-9

[b25] Waters J. H. & Dyga R. M. Postoperative blood salvage: outside the controlled world of the blood bank. Transfusion 47, 362 (2007). 17319811 10.1111/j.1537-2995.2007.01152.x

[b26] Thomassen B. J. *et al.* Limit allogeneic blood use with routine re-use of patient’s own blood: a prospective, randomized, controlled trial in total hip surgery. Plos One 7, e44503 (2012). 23028549 10.1371/journal.pone.0044503 PMC3441549

[b27] Cheung G. *et al.* No drain, autologous transfusion drain or suction drain? A randomised prospective study in total hip replacement surgery of 168 patients. Acta Orthop Belg 76, 619 (2010). 21138217

[b28] Atay E. F. *et al.* Allogeneic blood transfusion decreases with postoperative autotransfusion in hip and knee arthroplasty. Acta Orthop Traumatol Turc 44, 306 (2010). 21252608 10.3944/AOTT.2010.2417

[b29] Tripkovic B. *et al.* Quality of the blood sampled from surgical drainage after total hip arthroplasty. Coll Antropol 32, 153 (2008). 18494201

[b30] Slappendel R., Horstmann W., Dirksen R. & van Hellemondt G. G. Wound drainage with or without blood salvage? An open, prospective, randomized and single‐center comparison of blood loss, postoperative hemoglobin levels and allogeneic blood transfusions after major hip surgery. Transfus Altern Transfus Med 10, 174 (2008).

[b31] So-Osman C., Nelissen R. G., Eikenboom H. C. & Brand A. Efficacy, safety and user-friendliness of two devices for postoperative autologous shed red blood cell re-infusion in elective orthopaedic surgery patients: A randomized pilot study. Transfus Med 16, 321 (2006). 16999754 10.1111/j.1365-3148.2006.00705.x

[b32] Ayers D. C., Murray D. G. & Duerr D. M. Blood salvage after total hip arthroplasty. J Bone Joint Surg Am 77, 1347 (1995). 7673284 10.2106/00004623-199509000-00009

[b33] Slagis S. V., Benjamin J. B., Volz R. G. & Giordano G. F. Postoperative blood salvage in total hip and knee arthroplasty. A randomised controlled trial. J Bone Joint Surg Br 73, 591 (1991). 1906472 10.1302/0301-620X.73B4.1906472

[b34] Higgins J. P. & Green S. *Cochrane Handbook for Systematic Reviews of Interventions* Version 5.1.0. (2011). Available at: handbook.cochrane.org. (Accessed: 3rd March 2015).

[b35] Holst L. B., Petersen M. W., Haase N., Perner A. & Wetterslev J. Restrictive versus liberal transfusion strategy for red blood cell transfusion: systematic review of randomised trials with meta-analysis and trial sequential analysis. BMJ 350, h1354 (2015). 25805204 10.1136/bmj.h1354 PMC4372223

[b36] Teng Z. *et al.* Restrictive blood transfusion strategies and associated infection in orthopedic patients: a meta-analysis of 8 randomized controlled trials. Sci Rep 5, 13421 (2015). 26306601 10.1038/srep13421 PMC4549631

[b37] Dusik C. J., Hutchison C. & Langelier D. The merits of cell salvage in arthroplasty surgery: an overview. Can J Surg 57, 61 (2014). 24461268 10.1503/cjs.026612 PMC3908998

[b38] Rohde J. M. *et al.* Health care-associated infection after red blood cell transfusion: a systematic review and meta-analysis. JAMA 311, 1317 (2014). 24691607 10.1001/jama.2014.2726 PMC4289152

[b39] Gao F. *et al.* Topical Application of Tranexamic Acid Plus Diluted Epinephrine Reduces Postoperative Hidden Blood Loss in Total Hip Arthroplasty. J Arthroplasty 30, 2196 (2015). 26145190 10.1016/j.arth.2015.06.005

[b40] So-Osman C. *et al.* Patient blood management in elective total hip- and knee-replacement surgery (Part 1): a randomized controlled trial on erythropoietin and blood salvage as transfusion alternatives using a restrictive transfusion policy in erythropoietin-eligible patients. Anesthesiology 120, 839 (2014). 24424070 10.1097/ALN.0000000000000134

[b41] So-Osman C. *et al.* Patient blood management in elective total hip- and knee-replacement surgery (part 2): a randomized controlled trial on blood salvage as transfusion alternative using a restrictive transfusion policy in patients with a preoperative hemoglobin above 13 g/dl. Anesthesiology 120, 852 (2014). 24434302 10.1097/ALN.0000000000000135

[b42] Browne J. A., Adib F., Brown T. E. & Novicoff W. M. Transfusion rates are increasing following total hip arthroplasty: risk factors and outcomes. J Arthroplasty 28, 34 (2013). 23896359 10.1016/j.arth.2013.03.035

[b43] Frisch N. B. *et al.* Predictors and complications of blood transfusion in total hip and knee arthroplasty. J Arthroplasty 29, 189 (2014). 25007727 10.1016/j.arth.2014.03.048

[b44] van Bodegom-Vos L. *et al.* Cell Salvage in Hip and Knee Arthroplasty: A Meta-Analysis of Randomized Controlled Trials. J Bone Joint Surg Am 97, 1012 (2015). 26085536 10.2106/JBJS.N.00315

[b45] Park J. H. *et al.* Predictors of perioperative blood loss in total joint arthroplasty. J Bone Joint Surg Am 95, 1777 (2013). 24088970 10.2106/JBJS.L.01335

[b46] Goodnough L. T. & Shah N. Is there a “magic” hemoglobin number? Clinical decision support promoting restrictive blood transfusion practices. Am J Hematol 90, 927 (2015). 26113442 10.1002/ajh.24101

[b47] Carson J. L. *et al.* Red blood cell transfusion: a clinical practice guideline from the AABB*. Ann Intern Med 157, 49 (2012). 22751760 10.7326/0003-4819-157-1-201206190-00429

[b48] Conlon N. P., Bale E. P., Herbison G. P. & McCarroll M. Postoperative Anemia and Quality of Life After Primary Hip Arthroplasty in Patients Over 65 Years Old. Anesthesia & Analgesia 106, 1056 (2008). 18349173 10.1213/ane.0b013e318164f114

[b49] Newman J. H., Bowers M. & Murphy J. The clinical advantages of autologous transfusion. A randomized, controlled study after knee replacement. J Bone Joint Surg Br 79, 630 (1997). 9250753 10.1302/0301-620x.79b4.7272

[b50] Crescibene A., Martire F., Gigliotti P., Rende A. & Candela M. Postoperative Autologous Reinfusion in Total Knee Replacement. J Blood Transfus 2015, 826790 (2015). 26442168 10.1155/2015/826790 PMC4579317

[b51] Munoz M. *et al.* Transfusion of post-operative shed blood: laboratory characteristics and clinical utility. Eur Spine J 13, Suppl 1, S107 (2004). 15138860 10.1007/s00586-004-0718-0 PMC3592179

[b52] Vanderlinde E. S., Heal J. M. & Blumberg N. Autologous transfusion. BMJ 324, 772 (2002). 11923162 10.1136/bmj.324.7340.772 PMC1122708

[b53] Liumbruno G. M., Bennardello F., Lattanzio A., Piccoli P. & Rossetti G. Recommendations for the transfusion management of patients in the peri-operative period. III. The post-operative period. Blood Transfus 9, 320 (2011). 21627922 10.2450/2011.0076-10 PMC3136601

[b54] Liumbruno G. M. & Waters J. H. Unwashed shed blood: should we transfuse it? Blood Transfus 9, 241 (2011). 21627923 10.2450/2011.0109-10 PMC3136589

[b55] Hansen E. & Hansen M. P. Reasons against the retransfusion of unwashed wound blood. Transfusion 44, 45S (2004). 15585005 10.1111/j.0041-1132.2004.04179.x

[b56] Horstmann W. G., Slappendel R., van Hellemondt G. G., Castelein R. M. & Verheyen C. C. P. M. Safety of retransfusion of filtered shed blood in 1819 patients after total hip or knee arthroplasty. Transfus Altern Transfus Med 11, 57 (2010).

[b57] Hozo S. P., Djulbegovic B. & Hozo I. Estimating the mean and variance from the median, range, and the size of a sample. Bmc Med Res Methodol 5, 13 (2005). 15840177 10.1186/1471-2288-5-13 PMC1097734

